# Supplementary material for: Etiology of Clinical Community-Acquired Pneumonia in Swedish Children Aged 1–59 Months with High Pneumococcal Vaccine Coverage—The TREND Study
Source: Vaccines (Basel). 2021 Apr 14;9(4):384. doi: 10.3390/vaccines9040384 (PMC8070909; doi:10.3390/vaccines9040384)
Supplement: Supplementary file 1 [file vaccines-09-00384-s001.zip › vaccines-1146514-supplementary.pdf]

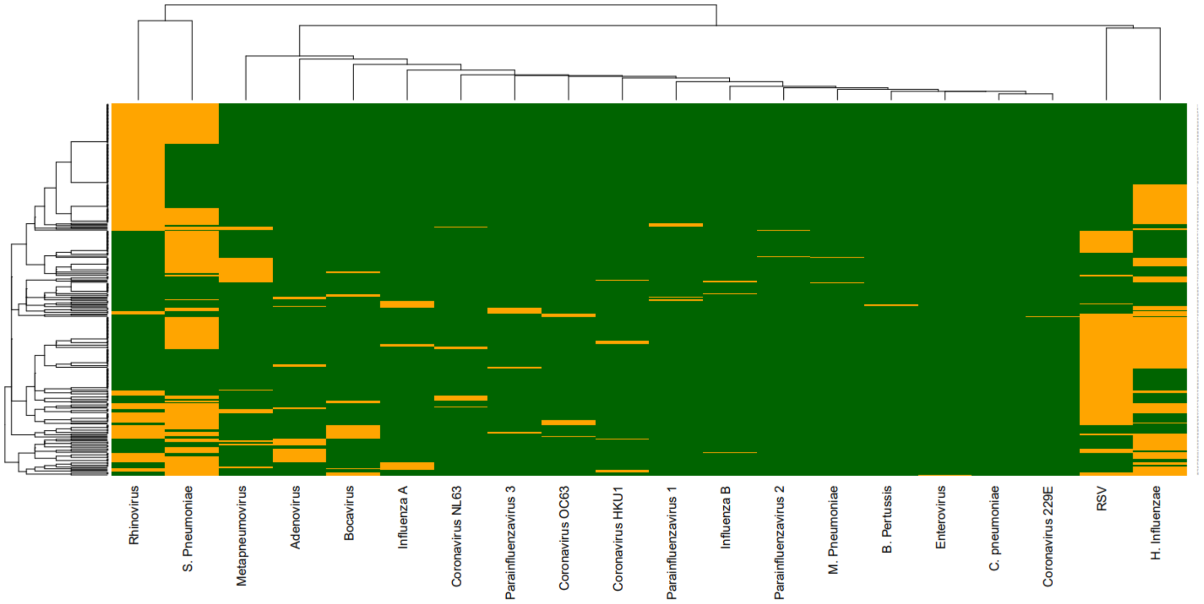

Figure S1 – Heatmap of respiratory real-time PCR-findings in children aged 1-59 months with clinical CAP

Euclidean hierarchical clustering of 327 patient cases (vertical axis) versus 20 PCR-tested pathogens (horizontal axis). An orange cell indicates a positive PCR test for a patient. For instance, in the top segment of rows, patients simultaneously positive for rhinovirus and *S. pneumoniae* are seen. The cluster map illustrates patterns of coinfections in groups of patients, as well as groups of frequently co-occurring pathogens, such as rhinovirus+*S. pneumoniae* (left) and RSV+*H. influenzae* (right).

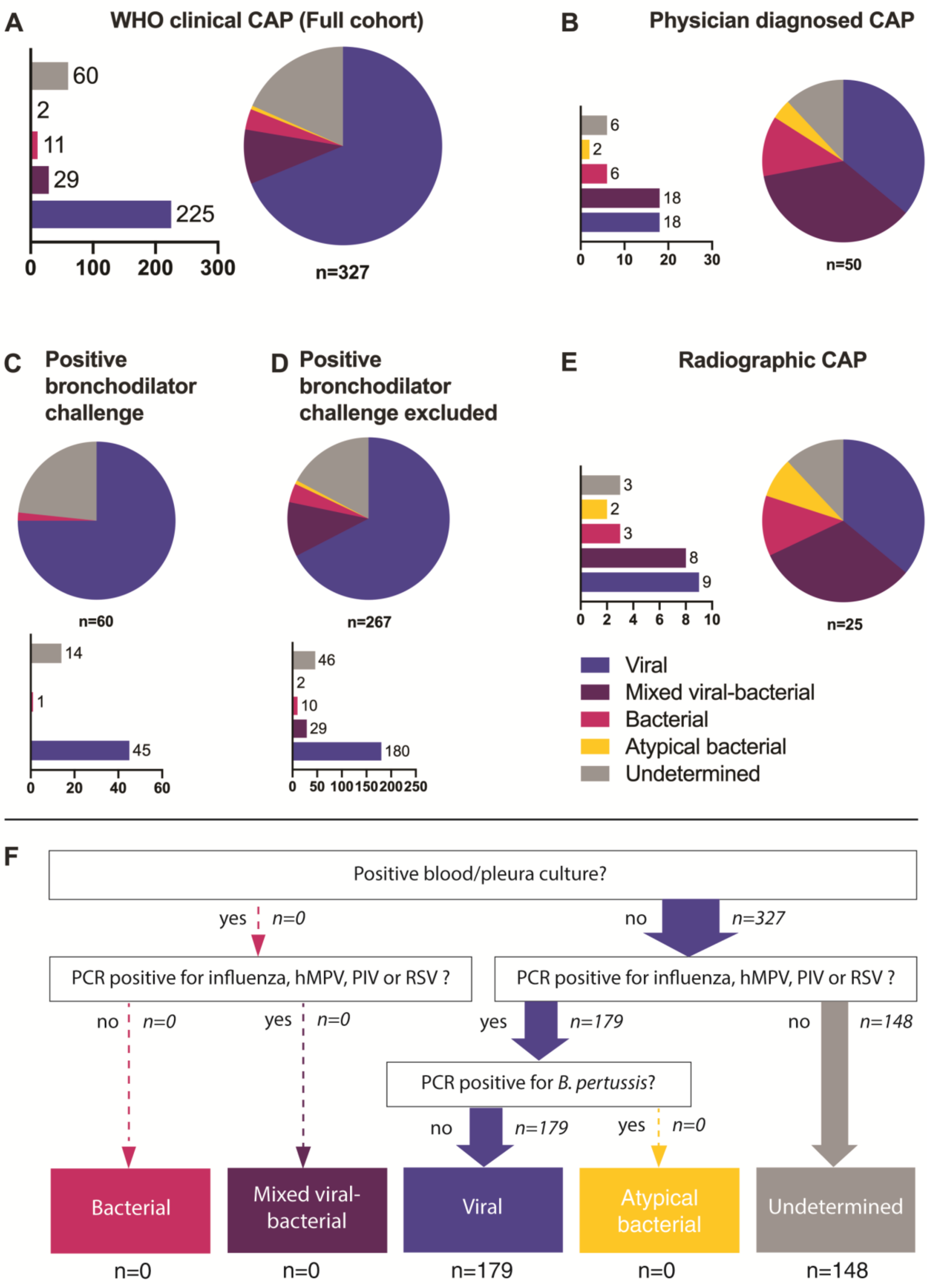

Figure S2. Etiology of children aged 1-59 months with clinical CAP in the TREND study varying the etiology algorithm. Etiology using CRP cutoff 60 mg/L for definition of bacterial etiology A) in the full cohort (n=327), B) in children with physician-diagnosed CAP (ICD-10 code of J10.0, J11.0 or J12-J18) (n=50), C) in children with (n=60) or D) without (n=268) positive bronchodilator challenge and E) in children with radiographic CAP (n=25). F) Etiology according to the strict TREND algorithm based on microbiologically confirmed diagnosis in the full cohort (n=328). Abbreviations: CAP, community-acquired pneumonia; WHO, world health organization.

Table S1. Characteristics of children responding to bronchodilator challenge

**Characteristic**	**Children responding to bronchodilator challenge** (n=60)	**Children not responding to bronchodilator challenge** (n=79)	**Children not receiving/not eligible for bronchodilator challenge** (n=188)	p-value
Age (months), median IQR	17 (10-26)	14 (7-23)	11 (3-20)	<0.001
Male sex	34 (57)	52 (66)	114 (61)	0.53
Attending pre-school	30 (52)	30 (38)	75 (41)	0.25
Breastfeeding	11 (19)	24 (30)	71 (38)	0.02
Parental smoking	9 (16)	13 (16)	23 (12)	0.62
Chronic disease	32 (56)	32 (41)	43 (24)	<0.001
Asthma	*31 (54)*	*29 (37)*	*31 (17)*	
Other	*1 (2)*	*3 (4)*	*12 (7)*	
University studies ≥1 parent	42 (70)	65 (82)	140 (74)	0.22
Clinical presentation				
Tachypnea[c]	56 (93)	70 (89)	165 (88)	0.53
Cough	58 (98)	79 (100)	176 (97)	0.32
Breathing troubles	44 (86)	58 (84)	120 (75)	0.11
History of fever	47 (85)	55 (77)	145 (80)	0.52
Peripheral oxygen saturation <90%	-	9 (12)	16 (9)	0.01
Nasal flaring	8 (13)	9 (12)	14 (8)	0.28
Grunting	2 (3)	9 (12)	11 (6)	0.13
CRP mg/L median (IQR)	10 (0-24)	13 (0-24)	15 (0-46)	0.18
PCR findings				
Adenovirus	1 (2)	5 (6)	18 (10)	0.11
Bocavirus	4 (7)	8 (10)	9 (5)	0.28
Coronavirus (any)	6 (10)	4 (5)	14 (7)	0.56
Enterovirus	-	-	1 (1)	1.0
Influenza virus (any)	2 (3)	1 (1)	15 (8)	0.06
Metapneumovirus	7 (12)	4 (5)	21 (11)	0.26
Parainfluenza virus	3 (5)	6 (8)	7 (4)	0.36
RSV	18 (30)	28 (35)	80 (43)	0.18
Rhinovirus	37 (62)	45 (57)	74 (39)	<0.01
B. pertussis	-	-	1 (1)	1.0
H. influenzae	26 (43)	28 (35)	89 (47)	0.20
M. pneumoniae	-	-	2 (1)	1.0
S. pneumoniae	34 (57)	37 (47)	108 (57)	0.27

[a] Age-adjusted (>50 breaths/min in children <1 year, >40 breaths/min in children 1-4 years). Abbreviations: IQR, interquartile range.

Table S2 - Coinfections. Total PCR-positive infections in the cohort were dominated by *S. pneumoniae*, Rhinovirus, *H. influenzae* and RSV. There were relatively few single infections, with the exception of 36 patients presenting with only Rhinovirus positivity. *S. pneumoniae*, Rhinovirus, *H. influenzae* and RSV also dominated among coinfections, with *S. pneumoniae*+*H. influenzae* (83), *S. pneumoniae*+Rhinovirus (83), *S. pneumoniae*+RSV (79), *H. influenzae* + RSV (66) and *H. influenzae* + Rhinovirus (59) being the most common pairwise coinfections.

Group	Adenovirus	Bocavirus	Coronavirus 229E	Coronavirus HKU1	Coronavirus NL63	Coronavirus OC63	Enterovirus	*H. influenzae*	Influenza B	Influenza A	Metapneumovirus	M. Pneumoniae	Parainfluenzavirus 1	Parainfluenzavirus 2	Parainfluenzavirus 3	*B. pertussis*	*S. pneumoniae*	Rhinovirus	RSV	*C. pneumoniae*
Total infections	24	21	1	7	9	8	1	144	3	15	32	2	6	2	8	1	179	156	127	0
Single infections	1	2	0	0	0	0	0	2	1	2	2	1	1	0	0	0	3	36	19	0
Coinfections	23	19	1	7	9	8	1	142	2	13	30	1	5	2	8	1	176	120	108	0
Adenovirus																				
Bocavirus	0																			
Coronavirus 229E	0	0																		
Coronavirus HKU1	1	0	0																	
Coronavirus NL63	0	0	0	0																
Coronavirus OC63	0	1	1	0	0															
Enterovirus	0	1	0	0	0	0														
H. influenzae	18	8	0	7	3	4	1													
Influenza B	1	0	0	0	0	0	0	2												
Influenza A	1	1	0	0	0	0	0	9	0											
Metapneumovirus	2	1	0	1	1	0	0	20	1	1										
M. pneumoniae	0	0	0	0	0	0	0	0	0	0	0									
Parainfluenzavirus 1	1	0	0	0	0	0	0	1	0	0	0	0								
Parainfluenzavirus 2	0	0	0	0	0	0	0	0	0	0	0	0	0							
Parainfluenzavirus 3	0	2	0	0	0	0	0	5	0	0	0	0	0	0						
B. pertussis	0	0	0	0	0	0	0	0	0	1	0	0	0	0	0					
S. pneumoniae	13	14	0	6	7	4	1	83	1	9	23	1	3	1	5	0				
Rhinovirus	9	13	0	1	2	4	0	59	0	2	4	0	3	1	4	0	83			
RSV	7	6	1	3	8	7	1	66	1	3	6	0	0	0	1	0	79	20		
C. pneumoniae	0	0	0	0	0	0	0	0	0	0	0	0	0	0	0	0	0	0	0	
